# Evaluation of Neonatal Hemolytic Jaundice: Clinical and Laboratory Parameters

**DOI:** 10.3889/oamjms.2015.129

**Published:** 2015-12-02

**Authors:** Anet Papazovska Cherepnalkovski, Vjekoslav Krzelj, Beti Zafirovska-Ivanovska, Todor Gruev, Josko Markic, Natasa Aluloska, Nikolina Zdraveska, Katica Piperkovska

**Affiliations:** 1 *University Pediatric Clinic - Neonatology, Faculty of Medicine, Ss Cyril and Methodius University of Skopje, Skopje, Republic of Macedonia*; 2 *University Hospital Split and School of Medicine, University of Split, Split, Croatia*; 3 *Institute of Epidemiology and Biostatistics, Faculty of Medicine, Ss Cyril and Methodius University of Skopje, Skopje, Republic of Macedonia*; 4 *Institute for Clinical Biochemistry, Faculty of Medicine, Ss Cyril and Methodius University of Skopje, Skopje, Republic of Macedonia*

**Keywords:** hemolytic jaundice, ABO/Rh isoimmunisation, unspecific etiology, laboratory

## Abstract

**BACKGROUND:**

Neonatal jaundice that occurs in ABO or Rhesus issoimunisation has been recognized as one of the major risk factors for development of severe hyperbilirubinemia and bilirubin neurotoxicity.

**AIM:**

Aim of our study was to investigate clinical and laboratory parameters associated with hemolytic jaundice due to Rh and ABO incompatibility and compare results with the group of unspecific jaundice.

**MATERIAL AND METHODS:**

One hundred sixty seven (167) neonatal hyperbilirubinemia cases were included in the study, 24.6% of which presented with ABO/Rhesus type hemolytic jaundice, and the rest with unspecific jaundice. Evaluation included: blood count, reticulocites, serum bilirubin, aminotransferases, blood grouping, and Coombs test, also the day of bilirubin peak, duration of the hyperbilirubinemia, and additional bilirubin measurements.

**RESULTS:**

We showed significantly lower mean values of hemoglobin, erythrocytes and hematocrit and significantly higher values of reticulocytes in the group of ABO/Rh incompatibility compared to the group of jaundice of unspecific etiology; also an earlier presentation and a higher-grade jaundice in this group.

**CONCLUSIONS:**

The laboratory profile in ABO/Rh isoimmunisation cases depicts hemolytic mechanism of jaundice. These cases carry a significant risk for early and severe hyperbilirubinemia and are eligible for neurodevelopmental follow-up. Hematological parameters and blood grouping are simple diagnostic methods that assist the etiological diagnosis of neonatal hyperbilirubinemia.

## Introduction

Neonatal jaundice is a common phenomenon during the first week of postnatal life affecting almost two thirds of term newborns [1, 2]. The mechanism of neonatal hyperbilirubinemia is multifactorial, comprising primarily processes that contribute to increased bilirubin load, or diminished bilirubin clearance [1-3]. The former may result from causes that potentiate bilirubin production and the enterohepatic circulation, whereas the latter may result from immature conjugative capacity, and/or impaired hepatic uptake or excretion [1-3]. It has been shown that the imbalance between bilirubin production and conjugation plays an important role in the mechanism of neonatal bilirubinemia [4]. Although both genetic and environmental factors may contribute to the development of neonatal hyperbilirubinemia, the importance of genetically determined conditions has been increasingly recognized [5-8].

Bilirubin overproduction that occurs in ABO, Rhesus or minor blood group incompatibilities with a positive direct antiglobulin test has been recognized as one of the major risk factors for development of severe hyperbilirubinemia in infants of 35 or more weeks’ gestation [9]. Isoimmune hemolytic disease has been included in the “neurotoxicity risk factors” list aimed to emphasizing main risk factors associated with brain damage in severe hyperbilirubinemia [10]. Lower phototherapy and exchange transfusion threshold levels are recommended in isoimmune hemolytic disease in order to prevent acute manifestations of bilirubin toxicity that might evolve into chronic and permanent neurological sequelae- kernicterus. Features of the latter include athetoid cerebral palsy, hearing loss, and intellectual handicaps, visual and dental problems [1-3, 9-11].

In a previous study we showed a prevalence of 14.43% of hemolysis in a group of infants with indirect hyperbilirubinemia in a two-year period. We also showed a high prevalence (44.37%) of jaundice of unidentified etiology; the group included: exaggerated physiological jaundice, early and late onset breast-milk jaundice, and no identifiable etiology [12].

The aim of our study was to investigate clinical and laboratory parameters associated with hemolytic jaundice due to Rh and ABO incompatibility and compare results with the group of unspecific jaundice with unidentifiable etiology.

## Material and Methods

The study group included 167 newborns treated with neonatal hyperbilirubinemia at the University Pediatric Clinic’s Neonatology Department in Skopje, Republic of Macedonia; 41 patient (24.6%) who presented with ABO or Rhesus type hemolytic disease of the newborn, and 126 patients (75.4%) who were treated with unspecific neonatal jaundice (Table 1). Medical records were reviewed extensively to exclude clinical signs and symptoms that could increase the extent of jaundice such as: cephalhaemathoma, bruising, ecchymoses, lacerations, sepsis, prematurity, hypothyroidism, CNS hemorrhage, intestinal atresia or stenosis, hypertrophyc pyloric stenosis, delayed meconium passage, G6PD deficiency and Down syndrome [3, 12, 13]. The following laboratory tests were performed: full blood count and smear, hemoglobin and hematocrit levels, reticulocite count, serum levels of total, indirect and direct bilirubin, serum aminotransferases- aspartat transaminase (AST) and alanin transaminase (ALT), glucose-6-phosphate dehydrogenase (G6PD) quantitative test, maternal and neonatal blood groups, direct antiglobulin (Coombs) test. The study also included evaluation of the day at which bilirubin level reached peak, duration of the hyperbilirubinemia, as well as the first and second control bilirubin measurement where clinically indicated. In some cases, tests such as C-reactive protein (CRP), blood, cerebrospinal fluid and other cultures, thyroid function tests as well as ultrasonography of the central nervous system and radiograms were performed to exclude other etiologies of jaundice [1-3, 9, 11-13]. Hemolytic etiology of jaundice was considered in cases of Coombs positive ABO or Rh blood group incompatibility. Undefined etiology of jaundice was defined as previously described [12].

**Table 1:** Study group

	Etiology group	Number of patients	Percentage (%)

1.	Hemolysis (ABO and Rh incompatibility)	41	24.6

2.	Unspecific jaundice	126	75.4

	Total	167	100

Full blood count was analyzed using the Sysmex K-4500 automated hematology analyzer (Minesota, USA); light microscopy was involved when analyzing blood smear and reticulocites. Total serum bilirubin and fractions were obtained using photometric chemistry analyzer Kodak Ectachem 250 (Rochester, NY) [14]. G6PD activity in erythrocytes was determined spectrophotometricaly. The rate of absorbance change was measured at 340 nm, due to the reduction of nicotinamide adenine dinucleotide phosphate (NADP) to NADPH when a sample was incubated with G6P (Humananalyser 3000, Germany). G6PD activity was calculated in relation to erythrocyte count. Commercially available kits (AMS U.K. Ltd, East Sussex, U.K.) were used. Values of 272±27 mU/10^9^ erythrocytes were considered normal, and results were interpreted as percentage of the normal G6PD activity [15, 16].

Statistical analyses were performed using the statistical package Statistical Package for the Social Sciences (SPSS) 17.0 for Windows (SPSS Inc., Chicago, IL, USA). Categorical variables were presented with absolute numbers and percentages whereas quantitative variables were presented with mean, standard deviation, minimum, maximum, median and rang. Testing of significance between groups regarding the analyzed parameters was performed with: Student t-test, and Mann-Whitney U test. The result was considered significant if probability value (p) was <0.05 and <0.01 for high significance. The study has been approved by an institutional Ethics Committee in accordance with the Declaration of Helsinki.

## Results

The subject group consisted of 167 patients with indirect neonatal hyperbilirubinemia including 41 patients (24.6%) with ABO or Rhesus type hemolytic disease of the newborn, and 126 patients (75.4%) with unspecific neonatal jaundice (Table 1).

Laboratory parameters that were analyzed from full blood count were: 1) Hemoglobin (Hb), 2) Erythrocytes (Er), and 3) Hematocrit (Hct).

In the group of newborns with ABO/Rh incompatibility significantly lower mean values of hemoglobin (p = 0.038), erythrocytes (p = 0.0023) and hematocrit (p = 0.037) were noted compared to the group of jaundice with unspecific etiology (Table 2).

**Table 2:** Analyses from full blood count

Groups	Descriptive Statistics				

	N	mean±SD	min-max	t-value, p
Hb (g/l)				

1.	41	155.02 ± 30.3	74 - 218	t=2.09 p=0.038*

2.	126	165.36 ± 26.5	105 - 224	

Er (x10^12^)				

1.	41	4.29 ± 0.8	2.05 – 5.81	t=3.09 p=0.0023*

2.	126	4.67 ± 0.6	3.27 – 6.58	

Htc (%)				

1.	41	41.35 ± 8.9	18.9 – 61.9	t=2.11 p=0.037*

2.	126	44.26 ± 7.2	28.4 – 64.6	

				

* p < 0.05; t- Student t test; N- number of patients; SD- standard deviation; p- probability value.

Mean reticulocyte count was highly significantly higher (p = 0.000036) in the group of ABO/Rh incompatibility (27.88 ± 26.4 vs. 11.94 ± 7.4). In this group of newborns, the jaundice appeared significantly earlier (p < 0.01) compared to the group of jaundice with unidentified etiology (2.63 ± 2.4 vs. 4.02 ± 2.5 days). Duration of the bilirubin peak was significantly longer (p = 0.036) in the group of unspecific jaundice (15.03 ± 25.7 vs. 10.22 ± 9.02 days) (Table 3).

**Table 3:** Analyses of bilirubin

Groups	Descriptive Statistics				

	N	mean±SD	median	min-max	t-value, p
Ret					

1.	41	27.88 ± 26.4	22.0	2 - 121	Z=4.13 p=0.000036**

2.	126	11.94 ± 7.4	11.0	1 - 39	

Day of bilirubin peak					

1.	41	2.63 ± 2.4	2.0	1 – 14	Z=5.78 p=0.000**

2.	126	4.02 ± 2.5	3.0	2 – 14	

Peak bilirubin level (μmol/l)					

1.	41	379.76 ± 133.5	364.0	158 – 801	Z=1.95 p=0.052 NS

2.	126	333.44 ± 91.1	324.0	107 - 598	

Duration of the bilirubin peak (days)					

1.	41	10.22 ± 9.02	6.0	1 – 37	Z=2.09 p=0.036*

2.	126	15.03 ± 25.7	9.0	2 - 279	

First control bilirubin (μmol/l)					

1.	40	274.2 ± 124.9	235.5	96 – 682	Z=1.87 p=0.062 NS

2.	112	227.39 ± 80.7	211.5	60 - 473	

Second control bilirubin (μmol/l)					

1.	24	227.46 ± 83.4	206.0	111 – 437	Z=0.76 p=0.448 NS

2.	48	221.92 ± 48.3	228.5	51 - 314	


* p < 0.05;

** p<0,01 Z (Mann-Whitney U test); N- number of patients; SD- standard deviation; p- probability value; NS- not significant.

Newborns from the ABO/Rh incompatibility group compared to the group of newborns with unspecific jaundice had insignificantly higher peak bilirubin level (p = 0.052), as well as insignificantly higher levels at the first (p = 0.062) and second (p=0.448) control bilirubin measurements (Table 3).

Levels of hepatic transaminases (AST and ALT) were not found to depend significantly on the etiology of jaundice (Table 4).

**Table 4:** Amynotransferases- AST and ALT

Groups	Descriptive Statistics					

	N	mean±SD	median	min-max	t-value, p
AST (U/l)					

1.	41	62.51 ± 47.8	51.0	22 – 317	Z=0.067 p=0.95 NS

2.	125	58.62 ± 32.2	52.0	21 - 218	

ALT (U/l)					

1.	41	33.09 ± 23.5	28	3 – 109	Z=0.19 p=0.85 NS

2.	125	32.27 ± 24.5	30	3 - 170	

Z (Mann-Whitney U test); N- number of patients; SD- standard deviation; p- probability value; NS- not significant.

Treatment of jaundice was performed according to current protocols with continuous phototherapy/and or exchange transfusion [1, 3, 9, 17-21]. The bulk of the cases 161 (96.4%) were managed conventionally using double surface blue light phototherapy lamps at wave length of 460 nm. Six patients (3.6% of the whole group, and 14.6% of the hemolytic etiology group) were treated with exchange transfusion (ECT) out of which 4 (two thirds) patients presented with ABO incompatibility, and the remaining 2 with Rh incompatibility.

## Discussion

In this study we aimed to investigate clinical and laboratory parameters associated with hemolytic jaundice due to Rh and ABO incompatibility and compare results with a group of infants with jaundice of unspecific origin. We proved statistically significant correlations of all relevant laboratory parameters between the two groups. In a previous study, we found a high prevalence of jaundice of undefined etiology (44.37%), ascribed to cases where despite intensive workout, no identifiable cause or contributing factor for jaundice could be found [12]. We speculated an imbalance between bilirubin production and conjugation to be the key concept of jaundice in this group based on lack of history, clinical and laboratory data that would indicate another mechanism of jaundice [4, 12]. This group was used as a reference for comparison of clinical and laboratory parameters. Blood group incompatibility induced haemolysis (either ABO or Rh) has been identified as one of the risk factor for both severe hyperbilirubinemia and bilirubin neurotoxicity in infants of 35 or more weeks’ gestation [9, 10]. Moreover, it has been postulated that synergistic effect of DAT positive isoimmune hemolytic disease and severe hyperbilirubinemia potentiate bilirubin-induced neurotoxicity [22]. Lower phototherapy and exchange transfusion threshold levels have been recommended in isoimmune hemolytic disease to prevent manifestations of bilirubin encephalopathy, also a pre-discharge risk assessment and early post-discharge follow up [9, 10, 17-19]. In a Turkish study, 19 out of 93 (20.43%) extreme hyperbilirubinemia patients were isoimmunised [23]. In two other studies ABO isoimmunization was reported as the most common cause of hyperbilirubinemia requiring ECT, reported rates were 38% and 27.8% respectively [24, 25]. ECT was performed in 14.6% of our isoimmunisation cases, 66.6% of which were attributable to ABO incompatibility, and the rest to Rh incompatibility. We included only Coombs positive cases in our study to avoid uncertainty in interpreting etiology. We did not include any G6PD deficient cases in the group of hemolytic etiology for the same reason. Although G6PD deficiency associated neonatal hyperbilirubinemia was traditionally considered hemolytic in origin, it was shown that inadequate bilirubin conjugation in the liver was the key component of neonatal jaundice in these patients [26, 27]. We found significantly lower mean values of hemoglobin, erythrocytes and hematocrit and highly significantly higher values of reticulocytes in the group of ABO/Rh incompatibility compared to the group of jaundice of unspecific etiology. This confirms that hemolysis is the main component of jaundice in this group of patients and is consistent with findings from other studies [13, 28]. In this group of newborns, the jaundice appeared significantly earlier compared to the group of jaundice with unidentified etiology (at median age of 2 days). Although peak bilirubin level in the hemolytic group was insignificantly higher compared to the other group, a clear tendency to earlier rise and higher values of serum bilirubin were noted. Duration of the bilirubin peak was significantly longer in the group of unspecific jaundice. We can speculate that this finding might reflect differences in response to phototherapy in both groups, nevertheless, other impacts such as different mechanisms of jaundice, dissimilar biological progress of jaundice or stringency to phototherapy cannot be excluded. Levels of hepatic transaminases (AST and ALT) were not found to depend significantly on the etiology of jaundice. Although analysis of transaminases is performed on a regular basis in our hospital practices when evaluating an infant for jaundice, it is of little clinical value in cases of unconjugated hyperbilirubinemia and cannot be readily recommended. According to an evidence-based review on neonatal hyperbilirubinemia, the majority of kernicterus cases occurred in infants with a bilirubin level higher than 20 mg/dL (342 umol/l) [29]. It is clear that our hemolysis cases with mean peak bilirubin levels of 379.8 ± 133.5 umol/l are eligible for the neurotoxic effects of the high bilirubinemia especially the ones towards the higher end of the spectrum (maximum of 801 umol/l) and are candidates for long-term neurodevelopmental follow-up. Therefore, clinicians’ awareness of potential treats and harms that might be associated with isoimmunisation is vital.

In conclusion, aboratory profile in ABO and Rh isoimmunisation cases depicts hemolytic mechanism of jaundice. This group of patients is associated with a significant risk for early and severe hyperbilirubinemia and is eligible for long-term neurodevelopmental follow-up. Hematological parameters together with blood grouping are simple diagnostic methods that assist the etiological diagnosis of neonatal hyperbilirubinemia.

## References

[1] (2010). Detection and treatment of neonatal jaundice-NICE guideline. Lancet.

[2] Porter ML, Dennis BL (2002). Hyperbilirubinemia in the term newborn. Am Fam Physician.

[3] Dennery PA, Seidman DS, Stevenson DK (2001). Neonatal hyperbilirubinemia. N Engl J Med.

[4] Kaplan M, Muraca M, Hammerman C (2002). Imbalance between production and conjugation of bilirubin: a fundamental concept in the mechanism of neonatal jaundice. Pediatrics.

[5] Watchko JF, Daood MJ, Biniwale M (2002). Understanding neonatal hyperbilirubinemia in the era of genomics. Semin Neonatol.

[6] Kaplan M, Hammerman C, Maisels MJ (2003). Bilirubin genetics for the nongeneticist: hereditary defects of neonatal bilirubin conjugation. Pediatrics.

[7] Watchko JF, Lin Z, Clark RH (2009). Complex multifactorial nature of significant hyperbilirubinemia in neonates. Pediatrics.

[8] Lin Z, Fontaine J, Watchko JF (2008). Coexpression of gene polymorphisms involved in bilirubin production and metabolism. Pediatrics.

[9] American Academy of Pediatrics, Subcommittee on Hyperbilirubinemia (2004). Management of hyperbilirubinemia in the newborn infant 35 or more weeks of gestation. Pediatrics.

[10] Maisels MJ, Bhutani VK, Bogen D (2009). Hyperbilirubinemia in the newborn infant >or =35 weeks’ gestation: an update with clarifications. Pediatrics.

[11] Bhutani VK, Johnson LH, Maisels MJ (2004). Kernicterus: epidemiologic strategies for its prevention through systems-based approaches. J Perinatol.

[12] Papazovska Cherepnalkovski A, Piperkova K, Palcevska Kocevska S (2015). Evaluation and management of neonatal indirect hyperbilirubinemia at the University Pediatric Clinic in Skopje, Republic of Macedonia Medicus.

[13] Koosha A, Rafizadeh B (2007). Evaluation of neonatal indirect hyperbilirubinaemia at Zanjan Province of Iran in 2001-2003: prevalence of glucose-6-phosphate dehydrogenase deficiency. Singapore Med J.

[14] Curme H, Rand RN (1997). Early history of Eastman Kodak Ektachem slides and instrumentation. Clinical Chemistry.

[15] Krzelj V, Zlodre S, Terzic J (2001). Prevalence of G-6-PD deficiency in the Croatian Adriatic Coast population. Arch Med Res.

[16] Beutler E, Blume KG, Kaplan JC (1977). International Committee for standardization in haematology: recommended methods for red cell enzyme analysis. Br J Haematol.

[17] Alkalay AL, Simmons CF (2005). Hyperbilirubinemia guidelines in newborn infants. Pediatrics.

[18] Bratlid D, Nakstad B, Hansen TW (2011). National guidelines for treatment of jaundice in the newborn. Acta Paediatr.

[19] Beeby P, Evans N Jaundice. Royal Prince Alfred Hospital Web site. RPA Newborn Care Guidelines.

[20] Maisels MJ, McDonagh AF (2008). Phototherapy for Neonatal Jaundice. N Engl J Med.

[21] Rennie JM, Sehgal A, De A, Kendall GS, Cole TJ (2009). Range of UK practice regarding thresholds for phototherapy and exchange transfusion in neonatal hyperbilirubinaemia. Arch Dis Child Fetal Neonatal Ed.

[22] Kaplan M, Bromiker R, Hammerman C (2014). Hyperbilirubinemia, hemolysis, and increased bilirubin neurotoxicity. Semin Perinatol.

[23] Tiker F, Gulcan H, Kilicdag H, Tarcan A, Gurakan B (2006). Extreme hyperbilirubinemia in newborn infants. Clin Pediatr (Phila).

[24] Davutoğlu M, Garipardiç M, Güler E, Karabiber H, Erhan D (2010). The etiology of severe neonatal hyperbilirubinemia and complications of exchange transfusion. Turk J Pediatr.

[25] Hakan N, Zenciroglu A, Aydin M (2014). Exchange transfusion for neonatal hyperbilirubinemia: an 8-year single center experience at a tertiary neonatal intensive care unit in Turkey. J Matern Fetal Neonatal Med.

[26] Kaplan M, Hammerman C (1998). Severe neonatal hyperbilirubinemia, a potential complication of glucose-6-phosphate dehydrogenase deficiency. Current Controversies in Perinatal Care III.

[27] Kaplan M, Muraca M, Hammerman C (1998). Bilirubin conjugation, reflected by conjugated bilirubin fractions, in glucose-6-phosphate dehydrogenase-deficient neonates: a determining factor in the pathogenesis of hyperbilirubinemia. Pediatrics.

[28] Mishra JP, Mishra J, Padhi RK, Mishra S, Manjareeka M (2014). Hematological profile in neonatal jaundice. J Basic Clin Physiol Pharmacol.

[29] Ip S, Chung M, Kulig J (2004). An evidence-based review of important issues concerning neonatal hyperbilirubinemia. Pediatrics.

